# Long-Term Stress and Concomitant Marijuana Smoke Exposure Affect Physiology, Behavior and Adult Hippocampal Neurogenesis

**DOI:** 10.3389/fphar.2018.00786

**Published:** 2018-07-23

**Authors:** Kitti Rusznák, Kata Csekő, Zsófia Varga, Dávid Csabai, Ágnes Bóna, Mátyás Mayer, Zsolt Kozma, Zsuzsanna Helyes, Boldizsár Czéh

**Affiliations:** ^1^Neurobiology of Stress Research Group, János Szentágothai Research Centre and Centre for Neuroscience, Pécs, Hungary; ^2^Molecular Pharmacology Research Group, János Szentágothai Research Centre and Centre for Neuroscience, Pécs, Hungary; ^3^Department of Pharmacology and Pharmacotherapy, University of Pécs Medical School, Pécs, Hungary; ^4^Department of Biochemistry and Medical Chemistry, University of Pécs Medical School, Pécs, Hungary; ^5^Department of Forensic Medicine, University of Pécs Medical School, Pécs, Hungary; ^6^Department of Laboratory Medicine, University of Pécs Medical School, Pécs, Hungary

**Keywords:** body weight, BrdU, *Cannabis sativa*, chronic stress, cognitive function, doublecortin, hippocampus, self-grooming

## Abstract

Marijuana is a widely used recreational drug with increasing legalization worldwide for medical purposes. Most experimental studies use either synthetic or plant-derived cannabinoids to investigate the effect of cannabinoids on anxiety and cognitive functions. The aim of this study was to mimic real life situations where young people smoke cannabis regularly to relax from everyday stress. Therefore, we exposed young adult male NMRI mice to daily stress and concomitant marijuana smoke for 2 months and investigated the consequences on physiology, behavior and adult hippocampal neurogenesis. Animals were restrained for 6-h/day for 5-days a week. During the stress, mice were exposed to cannabis smoke for 2 × 30 min/day. We burned 2 “joints” (2 × 0.8 g marijuana) per occasion in a whole body smoking chamber. Cannabinoid content of the smoke and urine samples was measured by HPLC and SFC-MS/MS. Body weight gain was recorded daily and we did unrestrained, whole body plethysmography to investigate pulmonary functions. The cognitive performance of the animals was evaluated by the novel object recognition and Y maze tests. Anxietyrelated spontaneous locomotor activity and self-grooming were assessed in the open field test (OFT). Adult neurogenesis was quantified post mortem in the hippocampal dentate gyrus. The proliferative activity of the precursor cells was detected by the use of the exogenous marker 5-bromo-20-deoxyuridine. Treatment effects on maturing neurons were studied by the examination of doublecortin-positive neurons. Both stress and cannabis exposure significantly reduced body weight gain. Cannabis smoke had no effect on pulmonary functions, but stress delayed the maturation of several lung functions. Neither stress, nor cannabis smoke affected the cognitive functioning of the animals. Results of the OFT revealed that cannabis had a mild anxiolytic effect and markedly increased self-grooming behavior. Stress blocked cell proliferation in the dentate gyrus, but cannabis had no effect on this parameter. Marijuana smoke however had a pronounced impact on doublecortin-positive neurons influencing their number, morphology and migration. In summary, we report here that long-term stress in combination with cannabis smoke exposure can alter several health-related measures, but the present experimental design could not reveal any interaction between these two treatment factors except for body weight gain.

## Introduction

Marijuana is the most widely used illicit drug, as about 2.5% of the world population consume cannabis regularly (UNODC, 2017). Cannabis is increasingly legalized worldwide for medical and recreational purposes (Carliner et al., 2017; EMCDDA, 2017; Webster, 2018), which results in an increasing consumption while the long-term consequences on health are not well understood (e.g., Volkow et al., 2014, 2016; Levine et al., 2017). Thus, it is important to investigate the outcomes of prolonged use.

Advocates argue that marijuana is a safe and natural alternative for the treatment of a variety of medical and mental health conditions, but ambiguous data are reported in the literature on the health risks imposed by chronic cannabis use. Cannabidiol (CBD), a major constituent of *Cannabis sativa* and several components of the endocannabinoid system are increasingly viewed as potential ‘druggable’ targets for the treatment for anxiety-related disorders (Blessing et al., 2015; Lee et al., 2017; Patel et al., 2017). Indeed, anxiety is among the top five medical symptoms for which North Americans report using medical marijuana while its anxiolytic effectiveness is still not well documented (Turna et al., 2017). The correlation between cannabis use and cognitive enhancement or impairment is also ambiguous. While numerous clinical and preclinical data suggest a strong correlation between marijuana exposure and impaired cognition, it does not conclusively demonstrate that cannabis consumption alone is sufficient to cause these deficits in humans (Broyd et al., 2016; Curran et al., 2016; Volkow et al., 2016; Levine et al., 2017). At the same time, there are reports on positive effect of cannabis use on various cognitive and executive functions (Osborne et al., 2017; Gruber et al., 2018; Tervo-Clemmens et al., 2018).

The consequences of prolonged marijuana inhalation on respiratory health and lung cancer is also debated (Gates et al., 2014; Martinasek et al., 2016; Chatkin et al., 2017; Stone et al., 2018). Similarly, there is conflicting data on the effect of cannabis use on body weight. Cannabis is known to stimulate appetite and potentially promote weight gain in patients suffering from human immunodeficiency virus or cancer, whereas, findings of the large epidemiological studies in the general population, consistently indicate that users of marijuana tend to have lower body mass indices (Sansone and Sansone, 2014).

Adult neurogenesis in the hippocampal dentate gyrus is a unique form of neuroplasticity that has received substantial attention during the recent years. Adult-born neurons in the dentate play an essential role in normal cognitive functioning and in specific forms of learning (Denny et al., 2014; Anacker and Hen, 2017). Stress is a potent inhibitor of the proliferative activity of the precursor cells and also blocks the survival of the newly generated neurons (Gould et al., 1997; Czéh et al., 2001, 2002; Cameron and Schoenfeld, 2018). In consequence, stress-induced disruption of adult neurogenesis may play a role in the development of various psychiatric disorders, including depression, anxiety, and schizophrenia (Santarelli et al., 2003; Snyder et al., 2011; Surget et al., 2011; Kim et al., 2012; Schoenfeld and Cameron, 2015).

Cannabinoid receptors are highly expressed in the hippocampus, and recent studies suggest that facilitation of the cannabinoid signaling in the hippocampus may prevent stress-induced behavioral changes (Campos et al., 2013; Scarante et al., 2017; Fogaça et al., 2018). Numerous studies investigated the effect of various cannabinoids on adult hippocampal neurogenesis (Prenderville et al., 2015), but these studies used either plant-derived extracts or synthetic cannabinoids. The results of these studies are also inconsistent. Some studies report on positive, stimulatory effect (Jiang et al., 2005; Palazuelos et al., 2006; Marchalant et al., 2009; Wolf et al., 2010; Rivera et al., 2011; Suliman et al., 2018), while others document negative, inhibitory effect on adult neurogenesis (Realini et al., 2011; Abboussi et al., 2014; Lee et al., 2014; Steel et al., 2014), or no effect at all (Kochman et al., 2006; Maćkowiak et al., 2007; Steel et al., 2014).

Most of the preclinical studies use either synthetic cannabinoid agents or cannabis extracts to investigate the physiological and behavioral consequences. Typically, animals are injected with various doses of the synthetic compound in an acute or chronic (10–14 days) treatment protocol. The aim of the present study was to mimic a real life situation where cannabis is smoked by adolescents on a daily basis to ease everyday stress. We used young adult mice and subjected them to daily stress over a 2-month period and during the stress exposure the animals were also exposed to cannabis smoke. To investigate the health risks of such a treatment protocol, we examined body weight gain, pulmonary functions, emotional responses and cognitive performance, as well as cellular changes in adult hippocampal neurogenesis. The *Cannabis sativa* used in this experiment was obtained from the local Hungarian Police Superintendancy. To determine the amount of tetrahydrocannabinol (Δ9-THC), cannabinol (CBN), and cannabidiol (CBD) content of our sample, we did an HPLC analysis of the marijuana smoke and metabolites of these compounds were measured in urine samples collected from the animals.

## Materials and Methods

### Animals

Young male NMRI mice, weighing 20 ± 3 g (4 weeks of age; *n* = 36) at the beginning of the experiment, were used. In mice, the sexual maturity, i.e., the first vaginal estrus starts during puberty at postnatal day 25–50 depending on the mouse strain (Drickamer, 1981). This indicates, that the mice enrolled in this experiment were at their “late puberty” when the stress and cannabis treatment protocol started. Animals were purchased from “Toxi-Coop” Toxicological Research Center Ltd. (Budapest, Hungary), and kept (group-housed) in a temperature and humidity-controlled animal facility and maintained on a standard 12 h light/dark cycle (lights on at 8 AM). Food and water were available *ad libitum* in the home cages.

### Ethical Considerations

The experimental procedures were carried out according to the 1998/XXVIII Act of the Hungarian Parliament on Animal Protection and Consideration Decree of Scientific Procedures of Animal Experiments (243/1988). The studies were approved by the Ethical Committee on Animal Research of the University of Pécs according to the Ethical Codex of Animal Experiments, and license was given (License No. BA02/2000-35/2016).

### Experimental Design

**FIGURE 1:**
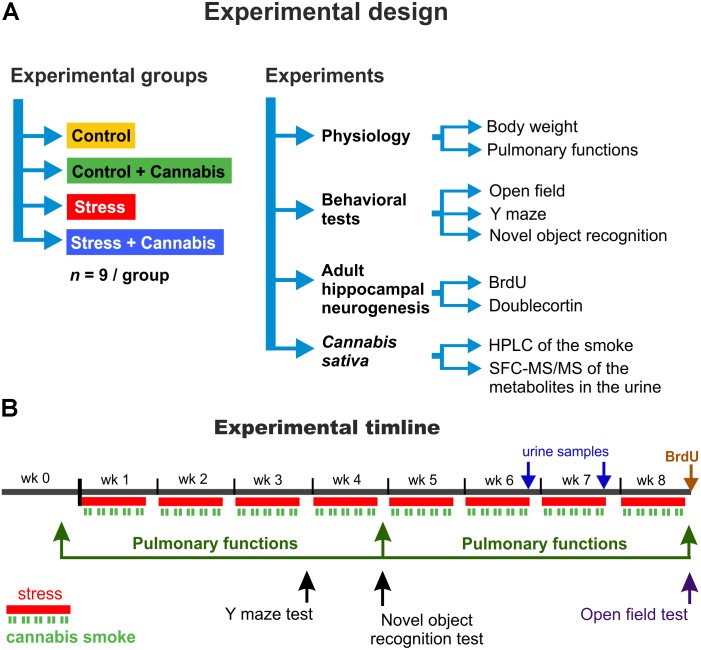
The experimental design and procedures. **(A)** Mice were divided into four experimental groups according to the stress and drug treatment protocol: Control, Control + Cannabis, Stress, and Stress + Cannabis (*n* = 9/experimental group). Various experimental tests and measurements were done to examine treatment effects on physiology, behavior and adult hippocampal neurogenesis. To determine the amount of Δ9-THC, CBD and CBN in our *Cannabis sativa* sample, we did a HPLC analysis of the marijuana smoke and SFC-MS/MS analysis was used to measure metabolites of these compounds in urine samples collected from the animals. **(B)** Timeline of the experimental procedures. First, the animals were allowed to habituate for 1 week. The long-term stress experiment lasted for 8 weeks. Animals were exposed to restraint stress every day for 6 h, 5-days a week (red lines). On weekends they had rest. During the restraint stress, mice were exposed to the cannabis smoke for 2 × 30 min/day (green lines). The cannabis smoke exposure was done at the end, i.e., during the last 2 h of the 6 h long restraint stress procedure and the smoke was applied simultaneously to all animals. We burned 2 × 2 “joints” (4 joints/day; 20 joints/week) in a manual smoking system and the animals had a whole body smoke exposure. All the behavioral tests were done on the weekends when the animals were not exposed to stress and/or marijuana smoke.

The experimental design including the animal groups, behavioral tests and the timeline of the procedures are depicted on **Figure 1**. First, the animals were allowed to habituate to the new housing conditions for 10 days. Afterward the animals were divided into four experimental groups according to the stress and drug treatment protocols: Control, Control + Cannabis, Stress, and Stress + Cannabis (*n* = 9/group). The long-term stress experiment lasted for 8 weeks. Animals were exposed to restraint stress every day, 5-days a week, but on the weekends they had rest. All the behavioral and the respiratory function tests were performed on weekends, when the animals were not subjected to stress or cannabis treatment. The behavioral testing started at 8:00 a.m. and measurement of one animal usually took 5–10 min. We tested the mice in an alternating order using a predefined sequence to minimize the potential effect of the circadian rhythm on the performance of mice from the different experimental groups.

### Restraint Stress Procedures

Animals were immobilized daily for 6 h, between 08:00 a.m. and 14:00 p.m. During restraint, the animals were placed in well ventilated polypropylene tubes (internal diameter, 3 cm; length, 11.5 cm) according to our pervious protocol (Scheich et al., 2017). During immobilization stress, mice did not have access to food or water. Control mice were not subjected to any kind of stress except daily handling. We used this restraint stress protocol because it has been demonstrated that this protocol is stressful for rodents and results in pronounced structural changes in the hippocampus, including dendritic atrophy and reduced adult neurogenesis in the dentate gyrus (McLaughlin et al., 2007; Veena et al., 2009; Yun et al., 2010).

### Chronic Whole Body Cannabis Smoke Exposure

Dried marijuana was provided by the Hungarian Police Superintendancy after the necessary permission was obtained from the Drug Licensing Department operating at the Office of Health Authorization and Administrative Procedures (Budapest, Hungary; License No. 15259/2016/KAB).

The marijuana was chopped, and cigarettes containing 0.77 ± 0.03 g marijuana were prepared. Mice were exposed to whole body marijuana smoke in a two-port TE-2 smoking chamber (TE-2 Teague Enterprises) from the age of 5 week for 8 weeks, twice a day for 30–30 min. Two joints were burned per occasion (4 joints/day; 20 joints/week; 154.97 ± 5.18 mg total particulate material/m^3^) for 10 min with a puff duration of 2 s and a puff frequency of 1/min/joint. This was followed by a 30 min long exposure period during which the smoke was driven into the chamber. After the smoke exposure there was a 20 min long ventilation period, during which the smoke was drawn out of the chamber by a vent hood, and then, the smoking session was repeated. Stressed mice were exposed to the cannabis smoke at the end, i.e., during the last 2 h of the 6 h long restraint stress procedure. Home cages containing the control animals, as well as mice subjected to the restraint stress were all placed in the smoking chamber simultaneously, therefore all mice were exposed to the marijuana smoke at the same time-point of their diurnal rhythm.

### HPLC Equipment and Chromatographic Conditions

Two cigarettes containing 0.77 ± 0.03 g marijuana were prepared and burned down in a smoking system. The formed marijuana smoke was pumped through a methanol containing gas-washing bottle by a vacuum pump. Methanol was removed by using a rotary evaporator. The sample was dissolved in 5 cm^3^ of methanol and filtered through a membrane filter (0.2 μm). The HPLC system used consisted of a Dionex P680 gradient pump (Dionex Corp., Sunnyvale, CA, United States), a helium degassing system, a Rheodyne 8125 injector valve with a 20 μL loop (Rheodyne Europe GmbH, Bensheim, Germany), and a Dionex 340D UV–vis diode array detector (Dionex Corp., Sunnyvale, CA, United States). The eluate was monitored at ambient temperature at different wavelengths, 209, 210, and 220 nm, respectively, where the investigated cannabinoids have their absorbance maxima. Chromatographic separations were achieved using Kinetex, C18 reversed phase column (2.6 μm, 2.1 mm × 150 mm i.d.). A Chromeleon data management software (Version 6.60 SP3 Build 1485, Dionex Corp., Sunnyvale, CA, United States) was used to control the equipment and for data evaluation. Peaks were identified by retention times and by UV spectrum of the respective compounds in comparison with those of the references. Quantification was carried out using the peak areas method. All measurements were made in triplicate.

The gradient consisted of two eluents: (A) water/methanol (90:10; v/v); (B) water/methanol (10:90; v/v) containing 50 mM of ammonium formate (adjusted to pH 5.20). The gradient profile was 0.0–20.0 min from 80% to 100% B, 20.0–25.0 min 100% B. After maintaining this condition for 5 min, the coloumn was set to initial condition in 1 min and re-equilibrated under this condition for 4 min. The total runtime was 30 min. Flow rate was set to 0.2 mL/min, the injection volume was 20 μL. All experiments were carried out at 25°C.

### Measurement of Cannabinoid Content of the Urine by SFC-MS/MS

Since cannabinoids have 97% plasma protein binding, penetrate to all tissues, and have high accumulation and urinary excretion (Nahas et al., 1981; Nahas and Latour, 1992), we determined the concentration of Δ9-THC, 11-nor-9-carboxy-THC, CBD and CBN in the urine. Urine samples (200–350 μl per mouse) were collected from all mice exposed to marijuana smoke by gently applying lower abdominal pressure on the 6th and 7th week of the experimental protocol (**Figure 1**). Samples were stored at 4°C and analyzed within 5 days.

After salting-out assisted liquid-liquid microextraction measurements were performed by an ACQUITY UPC^2^ supercritical fluid chromatography system (Waters) coupled with a Xevo TQ-S Triple Quadrupole Mass Spectrometer (Waters). Data were recorded by MassLynx software (V4.1 SCN950) and evaluated by TargetLynx XS software. Separation of compounds was performed on a 3.0 mm × 100 mm, 1.7 μm particle size, ACQUITY UPC^2^ Torus DIOL analytical column (Waters). The mobile phase consisted of the mixture of carbon dioxide (A) and 5 mM ammonium hydroxide in methanol (B) with flow rate of 1.2 mL/min. The following gradient profile was used: 99.9% A at 0.0 min and 82.0% A at 4.5 min. Pre-equilibration period lasting 2 min was applied before each injection. Constant 200 bar back pressure was used to maintain the supercritical state. The temperature was set at 45°C and the volume of injection was 1 μL. To sustain a suitable electrospray, additional solution consisted of 5 mM ammonium hydroxide in methanol was applied with flow rate of 0.1 mL/min. This makeup solvent was delivered by a Waters 515 HPLC Pump.

The MS measurement was performed in positive ion mode. The ESI source was operated with a spray voltage of 3.00 kV, cone voltage was 30 V. The source was set at 150°C. Both desolvation and cone gasses were nitrogen delivered at 300 and 150 L/min, respectively. Desolvation gas was temperatured at 300°C. Collision gas was argon with flow rate of 0.13 mL/min. MS/MS experiments were performed in MRM (multiple reaction monitoring) mode, monitoring five fragments with optimal collision energies.

### Pulmonary Function Tests

Pulmonary functions were measured by unrestrained whole body plethysmography individually in conscious, spontaneously breathing, freely moving animals repeatedly in a self-controlled manner. We did a baseline measurement on Week 0 (before the stress and cannabis exposure started) and subsequent measurements on Week 4 and Week 8 of the experimental procedures (**Figure 1**). Mice were placed in the chamber of a whole body plethysmograph (PLY 3211, Buxco Europe Ltd., Winchester, United Kingdom). The flow transducers (TRD5700, Buxco Europe Ltd., Winchester, United Kingdom) were connected to the preamplifier module, which digitized the signals via an analog-to-digital converter (MAX2270 Buxco Europe Ltd.). Ventilation parameters frequency (f), tidal volume (TV), time of inspiration (Ti), time of expiration (Te), minute ventilation (MV), peak inspiratory flow (PIF), and relaxation time (RT) were measured every 10 s for 2 min and averaged by the BioSystem XA Software for Windows (Buxco Research Systems). We also determined the enhanced pause (Penh) value, which is a calculated parameter [(expiratory time/relaxation time)-1]/(max. expiratory flow/max. inspiratory flow). This parameter characterize bronchoconstriction, which was induced by nebulizing 50 μl of 22 mM carbachol (a muscarinic acetylcholine receptor agonist). Acquisitions were taken in a 2-min-long baseline period aerosolized with saline and then 15 min after carbachol inhalation.

### Y Maze Test

The Y maze test was performed at the end of the 3rd experimental week (**Figure 1**) in an open Y-shaped maze placed on the ground with three 35 cm long gray plastic arms with 5 cm inner width (labeled A, B, and C) at 120° angle from each other lit from above. Mice were placed in the center of the maze and were allowed to freely explore the three arms for 5 min, their behavior was recorded with a video camera. Since mice generally prefer visiting a new arm of the maze and show a tendency to enter the less recently explored arm, the number of entries and triads (three consecutive visits of alternating arms) to calculate the spontaneous alternation were assessed afterward. Entries were counted when all four paws entered the arm.

### Novel Object Recognition Test

The novel object recognition test (NOR) was performed on the 4th week of the experimental procedures (**Figure 1**) in an open arena (45 × 45 cm with 30-cm-high surrounding walls) lit from above. The test lasted for 3 days: on the first day animals were habituated to the new environment for 10 min. On the second day two identical objects smaller than the mice were placed in the arena and mice were allowed to explore the objects for 5 min. On the last day, exactly 24 h later one of the identical objects was replaced with another object with different shape and color, and the behavior of the mice was recorded with a video camera for 5 min. The parameters of discrimination index [(time spent with novel object – time spent with familiar object)/time spent with both objects] and recognition index (time spent with novel object/time spent with both objects × 100) were assessed afterward.

### Open Field Test

The open field test (OFT) was performed at the end of the experimental procedures (**Figure 1**) in an open arena (45 × 45 cm with 30-cm-high surrounding walls) lit from above with a floor divided into 25 equal squares (9 × 9 cm). The test started by placing each mice individually into the center of the arena and then their behavior was recorded with a video camera for 5 min. The following parameters were analyzed afterward: time spent in the central fields (reciprocally proportional to the level of anxiety), locomotor activity (the time spent with moving), velocity (the ratio of the number of crossed squares/time spent with moving), time spent with grooming and grooming latency (time spent until the first grooming).

### BrdU Injection, Fixation and Post Mortem Processing of the Brain Tissue

The thymidine analog 5-bromo-2′-deoxyuridine (BrdU) was purchased from Sigma (Sigma–Aldrich, St. Louis, MO, United States). BrdU was dissolved in sterile 0.9% saline (containing 0.007 N NaOH) at a concentration of 15 mg/ml. To study cell proliferation, a single BrdU injection (200mg/kg, ip) was administered on the last experimental day. Animals were then perfused 24 h after the BrdU injection.

At the end of the experiment, mice were anesthetized deeply with a mixture of ketamine (Calypsol inj. 50 mg/ml, Richter Gedeon) and xylazine (Sedaxylan^®^ inj. 20 mg/ml, Eurovet Animal Health BV) 100/10 mg/kg administered intraperitoneally and then perfused transcardially with ice cold physiological saline, followed by freshly prepared ice cold 4% paraformaldehyde in 0.1 M phosphate buffer.

Brains were removed from the perfused animals, postfixed in perfusate overnight and sectioned on a Leica VT1200 S fully automated vibrating blade microtome. Serial coronal sections were cut throughout the entire hippocampal formation along the septo-temporal axis. The 50-μm sections were collected in series and stored in 0.1 M phosphate buffer (pH = 7.4) with 0.05% sodium azide at 4°C until staining.

### BrdU Immunocytochemistry

Every third section was slide-mounted on Superfrosts slides (Menzel-Glaser, Braunschweig, Germany), and coded to ensure objectivity before processing for immunocytochemistry. Immunohistochemistry was performed according to our standard protocol (Czéh et al., 2001, 2002, 2007). The sections were first washed in 0.1M Tris-buffer and then the DNA was denaturized in 0.1M citric acid at pH 6.0 and 95°C for 20 min. After treating the sections with 1% H_2_O_2_ in Tris for 20 min, the samples were washed in 0.1M Tris, and then, the cellular membranes were permeabilized with 0.1% trypsin in 0.1M Tris for 10 min. After thorough washing in Tris, the sections underwent acidification in 2N HCl in Tris for 30 min. After repeated rinsing in 0.1M Tris and PBS, non-specific binding was prevented by incubating the sections for 1h in 5% normal goat serum (NGS; Vector Laboratories, Burlingame, CA, United States) in PBS containing 0.5% Triton X-100 at 4°C. Subsequently, the sections were incubated for one night at 4°C with 1:5000 mouse anti-BrdU (DAKO, Clone Bu20a, Catalog # M074401) in the incubation solution (1% NGS, 0.5% Triton X-100 and 0,5% TWEEN-20 in PBS). After washing, the sections were incubated with biotinylated goat anti-mouse IgG (1:200, Vector Laboratories, Catalog # BA-9200) for 1 h at 4°C, thoroughly washed, incubated in avidin-biotin-horseradish peroxidase (1:500; Vectastaine Elite ABC Kit, Vector Laboratories) for 2 h at 4°C and then washed again. BrdU-labeled cells were visualized in 0.025% 3,30-diaminobenzidine (Sigma–Aldrich) and 0.01% H_2_O_2_ in PBS for 10 min. Sections were washed in PBS and counterstained with cresyl violet. After overnight drying at room temperature, the sections were dehydrated in graded alcohol, cleared in xylene and coverslipped with Eukitt.

### Doublecortin (DCX) Immunocytochemistry

Immunohistochemistry was performed according to our standard protocol (Csabai et al., 2016). Free-floating sections were washed in 0.1M PBS and then treated with 3% H_2_O_2_ for 30 min. After thorough washing in PBS, non-specific binding was prevented by incubating the sections for 1 h in 3% normal goat serum (NGS; Vector Laboratories) in PBS containing 0.5% Triton X-100. Subsequently, the sections were repeatedly rinsed in PBS for 1 h and incubated for one night at 4°C with a rabbit anti-DCX antibody (1:3000 Cell Signaling Technology Catalog # 4604). After thorough washing, the sections were incubated with anti-rabbit biotinylated secondary antibody (1:200; Vector Laboratories) for 2 h, washed and incubated in avidin-biotin-horseradish peroxidase (1:100; Vectastaine Elite ABC Kit, Vector) for 2 h. Labeled cells were visualized in 0.025% 3,30-diaminobenzidine (Sigma–Aldrich) and 0.01% H_2_O_2_ in PBS for 10 min. The sections were mounted, dried overnight and then dehydrated in graded alcohol, cleared and coverslipped with Eukitt. Slides were coded before quantification to ensure objectivity. Images were acquired on a Nikon Eclipse Ti-U workstation.

### Quantification of Adult Born Cells in the Dentate Gyrus

Cell were counted manually. A single experimenter (KR) who was blind to the group identification of each animal performed the data collection. The code was not broken until the cell counting analyses were completed. Cell counting was done using the Neurolucida (Version 7) reconstruction system (Microbrightfield, Colchester, VT, United States) attached to a Nikon Eclipse bright field microscope.

The quantitative analysis was carried out using a modified unbiased stereology protocol that has been reported to successfully quantify adult-born neurons in the dentate gyrus (Eisch et al., 2000; Czéh et al., 2001, 2002; Csabai et al., 2016). The BrdU+ and DCX+ cells were counted in a systematic manner in complete series of 50 μm thick sections starting at a random position along the entire septo-temporal axis of the hippocampal formation (from -0.94 to -3.88 relative to Bregma, according to the atlas of Franklin and Paxinos (2008). Every third section throughout the dentate gyrus was examined, yielding a mean of 25 sections per animal. All BrdU+ and DCX-labeled cells in the granule cell layer together with the subgranular zone, defined as a two-cell-body-wide zone along the border of the granule cell layer, were counted regardless of size or shape. Cells were examined under ×200 magnification, omitting cells in the outermost focal plane. The total number of BrdU+ or DCX+ cells was estimated by multiplying the number of cells counted in every third section by three. Neuron numbers are reported here as total neuron number/hemisphere.

DCX+ neurons were quantified similarly to the BrdU+ cells as described earlier (Csabai et al., 2016). We not only counted DCX+ cells, but also analyzed their morphology and cell migration. To examine cell migration, the neurons were selectively counted in the hilus, in the subgranular zone (sgz) and in the granule cell layer (gcl). The ratio of cells located in these three different cellular layers was expressed in percentage. Furthermore, we also counted the number of ectopic DCX+ neurons, i.e., cells which migrated out of the gcl into the molecular layer of the dentate gyrus. The morphological features of the DCX+ cells were quantified in two different ways. First, we did a semi-quantitative analysis in the following manner: we analyzed every 3rd serial section from each animal yielding 25 ± 3 sections/animal. After thorough visual inspection, every section was marked in which we observed abnormal looking DCX+ neurons (i.e., cells with irregular, disarrayed dendritic arbor). Data are reported as the number of sections including cells with abnormal morphology/animal. Afterward, we quantified the DCX+ neurons which had the most pronounced atypical appearance, i.e., bipolar DCX+ cells and DCX+ neurons with basal dendrites.

### Statistical Analysis

Results are expressed as the mean ± SEM. The data on body weights and pulmonary function tests were analyzed with three-way ANOVA (time × stress × cannabis treatment) followed by Tukey’s multiple comparisons *post hoc* test. The rest of the data was analyzed with two-way ANOVA (stress × cannabis treatment) followed by Tukey’s multiple comparisons *post hoc* test.

## Results

### Analysis of the Marijuana Smoke and Urine Samples

We performed a HPLC analysis to determine the Δ9-THC, CBD and CBN content of the marijuana smoke used in this study. Furthermore, we collected urine samples from the mice that were exposed to marijuana smoke on the 6th and 7th week of the experimental protocol (**Figure 1**). Concentration of Δ9-THC, 11-nor-9-carboxy-THC, CBD and CBN was determined by SFC/MS-MS in the urine. Data are reported in **Table 1**. The measurements revealed that the marijuana smoke had about 10× higher concentration of Δ9-THC than CBD. This ratio was even higher (30×) in the urine samples. The amount of 11-nor-9-carboxy-THC (a major metabolite of Δ9-THC) was 1.8 ± 0.2 in the urine (**Table 1**).

**Table 1:** Cannabinoid concentrations in the marijuana smoke and urine samples.

	Δ9-THC	CBD	CBN	11-nor-9-carboxy-THC
Marijuana smoke^∗^ (mg/mL)	0.427 ± 0.002	0.0358 ± 0.0011	0.258 ± 0.008	–
Urine samples (ng/mL)	65.6 ± 22.8	2.4 ± 0.6	41.9 ± 17.6	1.8 ± 0.2

^∗^*Chemical content of the smoke produced by burning 2 “joints” (2 × 0.77 g) of marijuana. Data are reported as mean ± SEM.*

### Stress and Cannabis Exposure Reduced the Body Weight Gain of the Animals

**FIGURE 2:**
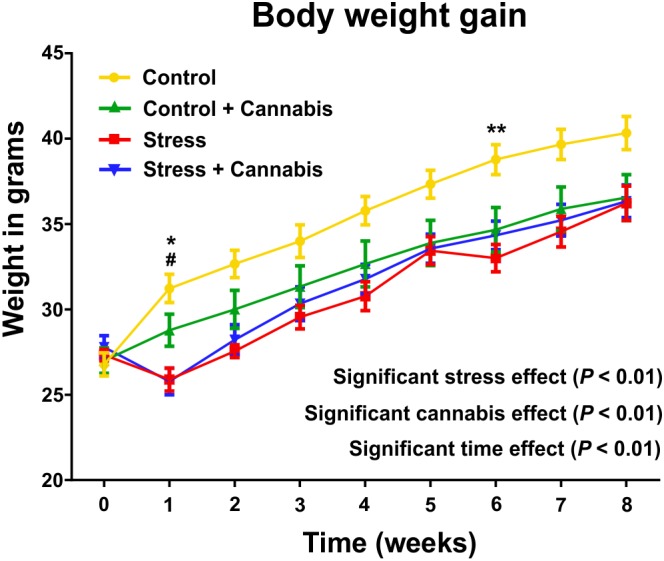
Repeated stress and cannabis exposure reduced body weight gain of the animals. Body weights were measured every day and then, the daily values were grouped and reported here as weekly means. Statistics: three-way ANOVA (time × stress × cannabis treatment) followed by Tukey’s multiple comparisons *post hoc* test. All three factors had highly significant main effect (*P* < 0.01) and there were significant interactions between time and cannabis treatment as well as between the stress and cannabis treatment. This indicates that cannabis treatment increasingly inhibited body weight gain as the animals grew and that cannabis smoke consumption could alleviate the stress-induced inhibitory effect on body weight gain. Tukey’s multiple comparisons revealed further group differences at specific time points: Control *versus* Stress group: ^∗^*P* < 0.05, ^∗∗^*P* < 0.01; Control *versus* Stress + Cannabis group: #*P* < 0.05.

Body weights were determined every day and then, the daily values were grouped and reported here as mean body weight/week (**Figure 2**). Analysis with a three-way ANOVA (time × stress × cannabis treatment) revealed that all three factors had a significant main effect: time: *F*(8,8) = 62.48, *P* < 0.001; stress: *F*(1,8) = 13.81, *P* < 0.002; and cannabis treatment: *F*(1,8) = 66.71, *P* < 0.001. Furthermore, there were significant interactions between the following two factors: time × cannabis treatment: *F*(8,8) = 2.11, *P* < 0.001 and stress × cannabis treatment: *F*(1,8) = 30.3, *P* < 0.01. This indicates that cannabis treatment increasingly inhibited body weight gain as the animals grew and that cannabis smoke consumption could alleviate the stress-induced inhibitory effect on body weight gain. For the results of the *post hoc* comparisons on group differences please see **Figure 2**.

### Stress Hindered the Maturation of Several Pulmonary Functions, but Marijuana Smoke Did Not Affect Lung Functions

**FIGURE 3:**
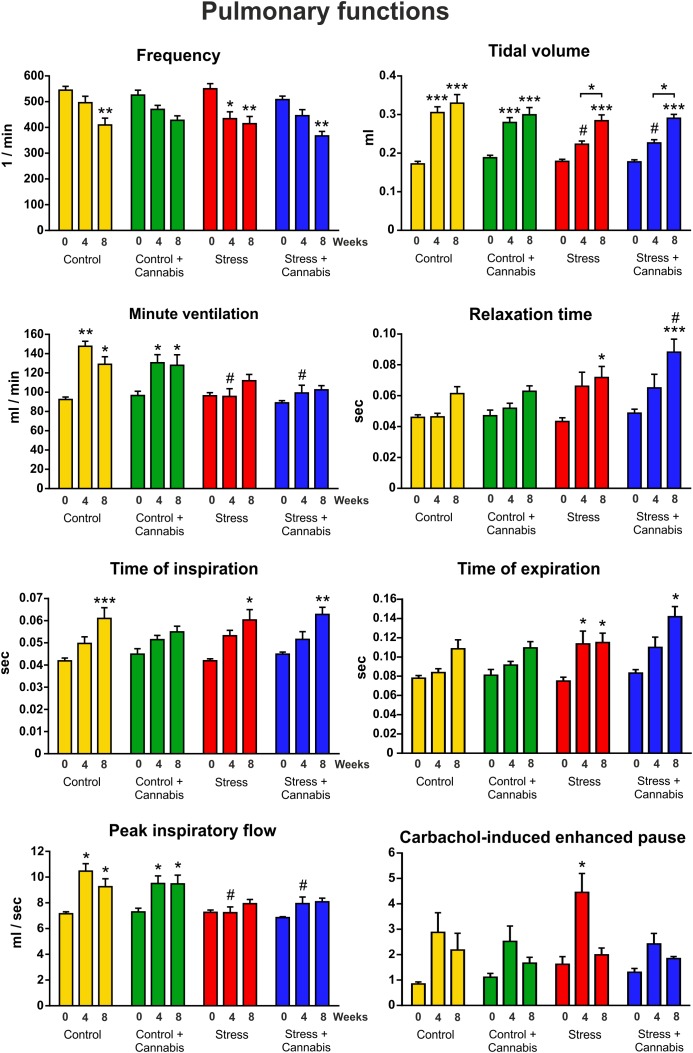
The effect of repeated stress and cannabis exposure on pulmonary functions. We did repetitive whole body plethysmography measurements on freely moving animals. Baseline values were recorded on Week 0, before the stress and cannabis exposure started and then subsequent measurements were done on Week 4 and Week 8 of the experimental procedures. Time (age) had the most pronounced effect on lung functions. As the animals grew bigger almost all values increased. Chronic stress significantly blocked the maturation of several lung parameters (minute ventilation, peak inspiratory flow and tidal volume) whereas, the relaxation time and the time of expiration were increased. Results of the three-way ANOVA (time × stress × cannabis treatment) showed a significant main effect of stress and significant time × stress interaction for these parameters. Marijuana smoke exposure affected only the carbachol-induced enhanced pause in breathing test. Tukey’s multiple comparisons revealed further group differences: ^∗^*P* < 0.05, ^∗∗^*P* < 0.01, ^∗∗∗^*P* < 0.001 compared to the value of Week 0 of the same treatment group. #*P* < 0.05 *versus* the Control group at the same experimental week.

We did repetitive lung plethysmography measurements in order to examine the effect of long-term stress and cannabis treatment on pulmonary functions. We hypothesized that long-term cannabis smoke exposure might affect lung functions. A baseline measurement was done on Week 0 (before the stress and cannabis exposure started) and subsequent measurements were done on Week 4 and Week 8 of the experimental procedures (**Figure 1**). Results of the plethysmography measurements are presented on **Figure 3**. Data were analyzed with a three-way ANOVA (time × stress × cannabis treatment) followed by Tukey’s multiple comparisons *post hoc* test. The main effects of the three-way ANOVA are presented in **Table 2**. Time (age) had the most pronounced effect on the pulmonary functional parameters. As the animals grew bigger all values of the pulmonary functions increased, except inhalation frequency, which decreased during maturation. Chronic stress altered the development of several lung parameters, i.e., the frequency, minute ventilation, peak inspiratory flow, time of expiration, relaxation time and tidal volume. For these parameters the three-way ANOVA showed a significant main effect of stress as well a significant time × stress interaction (**Table 2**). Chronic marijuana smoke exposure had no effect on any of the pulmonary functions except the carbachol-induced enhanced pause in breathing test (**Table 2**). The carbachol-induced enhanced pause is a functional parameter for the analysis of the bronchial hyper-reactivity of the lungs, correlating to the extent of airway inflammation. In this test bronchoconstriction is provoked by 50 μl nebulized carbachol (22 mM).

**Table 2:** Summary of the three-way ANOVA analysis (time × stress × cannabis treatment) of the pulmonary functional tests.

	Time (age)		Stress		Cannabis treatment		Interaction (time × stress)	
*f*	*F* = 35.95	*P* < 0.001	*F* = 4.38	*P* < 0.05	n.s.	n.s.	n.s.	n.s.
MV	*F* = 18.94	*P* < 0.001	*F* = 33.1	*P* < 0.001	n.s.	n.s.	*F* = 9.35	*P* < 0.001
Penh	*F* = 17.38	*P* < 0.001	n.s.	n.s.	*F* = 3.99	*P* < 0.05	n.s.	n.s.
PIF	*F* = 17.55	*P* < 0.001	*F* = 26.24	*P* < 0.001	n.s.	n.s.	*F* = 6.45	*P* < 0.01
RT	*F* = 20.50	*P* < 0.001	*F* = 12.71	*P* < 0.001	n.s.	n.s.	*F* = 3.46	*P* < 0.05
Te	*F* = 25.69	*P* < 0.001	*F* = 10.28	*P* < 0.01	n.s.	n.s.	n.s.	n.s.
Ti	*F* = 31.44	*P* < 0.001	n.s.	n.s.	n.s.	n.s.	n.s.	n.s.
TV	*F* = 100	*P* < 0.001	*F* = 20.33	*P* < 0.001	n.s.	n.s.	*F* = 7.16	*P* < 0.01

*Only the main effects of the three-way ANOVA are presented here. The results of the pulmonary functional tests are shown on **Figure 3**. f, frequency; MV, minute ventilation; n.s., non-significant; Penh, enhanced pause (induced by 22 mM carbachol); PIF, peak inspiratory flow; RT, relaxation time; Te, time of expiration; Ti, time of inspiration; TV, tidal volume.*

### Neither Stress, nor Marijuana Exposure Influenced Cognitive Behavior

**FIGURE 4:**
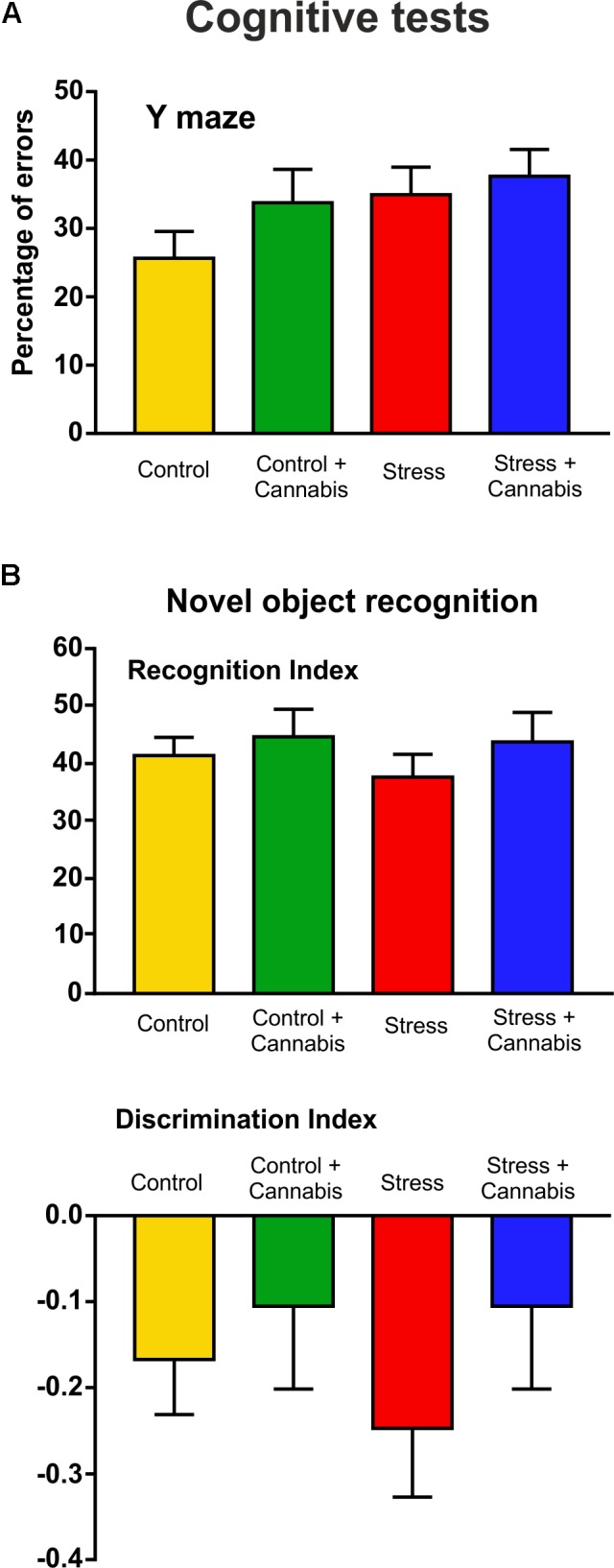
Results of the cognitive tests: Neither stress, nor marijuana exposure affected the cognitive abilities of the animals. Cognitive performances were measured in the Y maze **(A)** and with the novel object recognition test **(B)**. **(A)** Stressed and/or cannabis treated mice tended to make more errors in the Y maze test, i.e., they entered to arms which have been recently visited, but statistically there was no difference between the groups. **(B)** The recognition indexes (time spent with novel object/time spent with both objects × 100) were also similar for all experimental groups. Marijuana treated animals had the tendency to have a lower discrimination index [(time spent with novel object – time spent with familiar object)/time spent with both objects], but statistically this was not significant. Data were analyzed with a two-way ANOVA (stress × cannabis treatment).

The cognitive performance of the animals was evaluated using two different learning tests. We did a Y maze test at the end of Week 3 and a novel object recognition test at the end of Week 4 (**Figure 1**). Results of the cognitive tests are presented on **Figure 4**. At these time points, neither stress, nor marijuana exposure had any effect on the cognitive performance of the animals. In the Y maze, both the stressed and/or cannabis treated mice tended to make more errors, i.e., they entered to arms which have been recently visited, but statistically there were no difference between the groups (**Figure 4A**). In the novel object recognition test, the recognition index (time spent with novel object/time spent with both objects × 100) was similar for all experimental groups (**Figure 4B**). The cannabis treated animals had the tendency to have a lower discrimination index [(time spent with novel object – time spent with familiar object)/time spent with both objects], however, statistically this was not significant either (**Figure 4A**).

### Anxiety-Related Spontaneous Locomotor Activity and Self-Grooming in the Open Field Test

**FIGURE 5:**
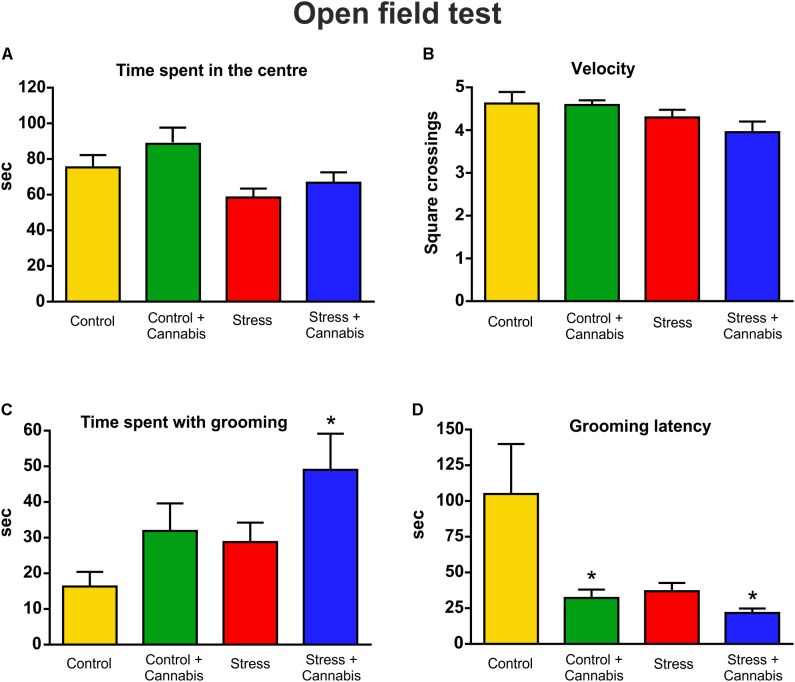
Results of the open field test. We quantified anxiety-related spontaneous locomotor activity and self-grooming in the open field test. **(A)** Stressed mice spent significantly less time in the center of the arena. Two-way ANOVA (stress × cannabis treatment) revealed a significant main effect of stress (*P* < 0.05), but Tukey’s *post hoc* test found no further group differences. Cannabis treated mice tended to spend more time in the center of the arena, but statistically this was not significant. **(B)** The velocity of the animals was evaluated by counting the number of squares that were crossed during the time spent with movement. Stressed mice had reduced exploratory activity. Two-way ANOVA (stress × cannabis treatment) yielded a significant main effect of stress (*P* < 0.05). Cannabis had no effect. **(C)** Cannabis had a pronounced effect on self-grooming. Both the stressed and cannabis treated mice spent significantly more time with self-grooming. Two-way ANOVA (stress × cannabis treatment) revealed a significant main effect of stress (*P* < 0.05), and of cannabis treatment (*P* < 0.05). Tukey’s *post hoc* test revealed a significant difference between the Control and Stress + Cannabis groups (^∗^*P* < 0.05). **(D)** Stressed and/or cannabis treated mice started to groom themselves significantly sooner than control mice. Two-way ANOVA (stress × cannabis treatment) revealed a significant main effect of stress (*P* < 0.05), and of cannabis treatment (*P* < 0.05). Tukey’s *post hoc* test revealed further significant differences between the Control and Control + Cannabis groups and between the Control and Stress + Cannabis treated groups (^∗^*P* < 0.05).

We performed an OFT at the end of the experiment to evaluate the anxiety-related spontaneous locomotor activity and self-grooming behavior of the animals (**Figure 1**). Stressed mice spent less time in the center of the arena (**Figure 5A**). Two-way ANOVA (stress × cannabis treatment) revealed a significant main effect of stress [*F*(1,32) = 6.33, *P* < 0.05], but Tukey’s *post hoc* test found no further group differences. Measurement of the velocity of the animals, i.e., the number of squares crossed during the time spent with movement, also revealed that stressed mice showed significantly reduced exploratory activity (**Figure 5B**). Two-way ANOVA (stress × cannabis treatment) yielded a significant main effect of stress [*F*(1,32) = 5.07, *P* < 0.05]. Cannabis treatment had no effect on these parameters, although animals exposed to marijuana smoke tended to spend more time in the center of the arena. However, cannabis treatment had a pronounced effect on self-grooming behavior (**Figures 5C,D**). Both the stressed and cannabis treated mice spent significantly more time with grooming. Two-way ANOVA (stress × cannabis treatment) revealed a significant main effect of stress [*F*(1,32) = 4.17, *P* < 0.05], and a significant main effect of cannabis treatment [*F*(1,32) = 6.11, *P* < 0.05], but no interaction between the two factors. Furthermore, Tukey’s multiple comparisons *post hoc* test revealed a significant difference between the Control and Stress + Cannabis treated groups (*q* = 4.51, *P* < 0.05) (**Figure 5C**). In addition to that, stressed and/or cannabis treated mice started the self-grooming much earlier than control mice (**Figure 5D**). We measured grooming latency and two-way ANOVA (stress × cannabis treatment) revealed a significant main effect of stress [*F*(1,32) = 4.71, *P* < 0.05], and a significant main effect of cannabis treatment [*F*(1,32) = 6.89, *P* < 0.05], but no interaction between the two factors. Tukey’s *post hoc* test revealed a significant difference between the Control and Control + Cannabis groups (*q* = 4.02, *P* < 0.05), as well as between the Control and Stress + Cannabis treated groups (*q* = 4.60, *P* < 0.05) (**Figure 5D**).

### The Stress-Induced Inhibition of Cell Proliferation in the Dentate Gyrus Was Not Influenced by Cannabis Smoke Exposure

**FIGURE 6:**
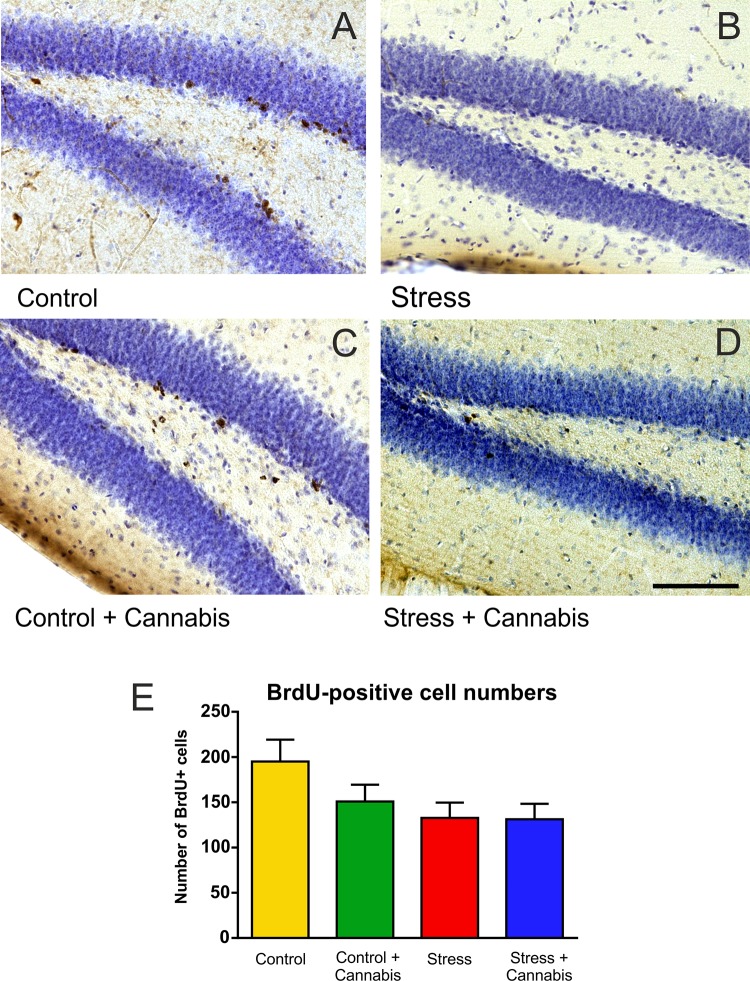
Stress inhibited progenitor cell proliferation in the dentate gyrus. Representative images demonstrating BrdU-immunohistochemistry labeling in the dentate gyrus of the animals from the different treatment groups **(A–D)**. **(E)** The total number of BrdU-positive cells in the dentate gyrus of one hemisphere. Stress significantly reduced the number of BrdU-positive cells in the dentate gyrus. Two-way ANOVA (stress × cannabis treatment) revealed a significant main effect of stress (*P* < 0.05), but cannabis treatment had no statistically significant effect on cell proliferation. Scale bar: 100 μm for all images.

Cell proliferation in the dentate gyrus was visualized with use of the exogenous proliferation marker BrdU (**Figures 6A–D**). Stress significantly reduced the number of BrdU-positive cells in the dentate gyrus (**Figure 6E**). Two-way ANOVA (stress × cannabis treatment) revealed a significant main effect of stress [*F*(1,32) = 4.43, *P* < 0.05]. The number of BrdU-positive cells were also fewer in the dentate gyrus of mice of the Control + Cannabis group, but cannabis treatment had no statistically significant effect on cell proliferation.

### Cannabis Smoke Exposure Had a Pronounced Effect on the Number, Morphology and Migration of the Doublecortin-Positive Immature Neurons

**FIGURE 7:**
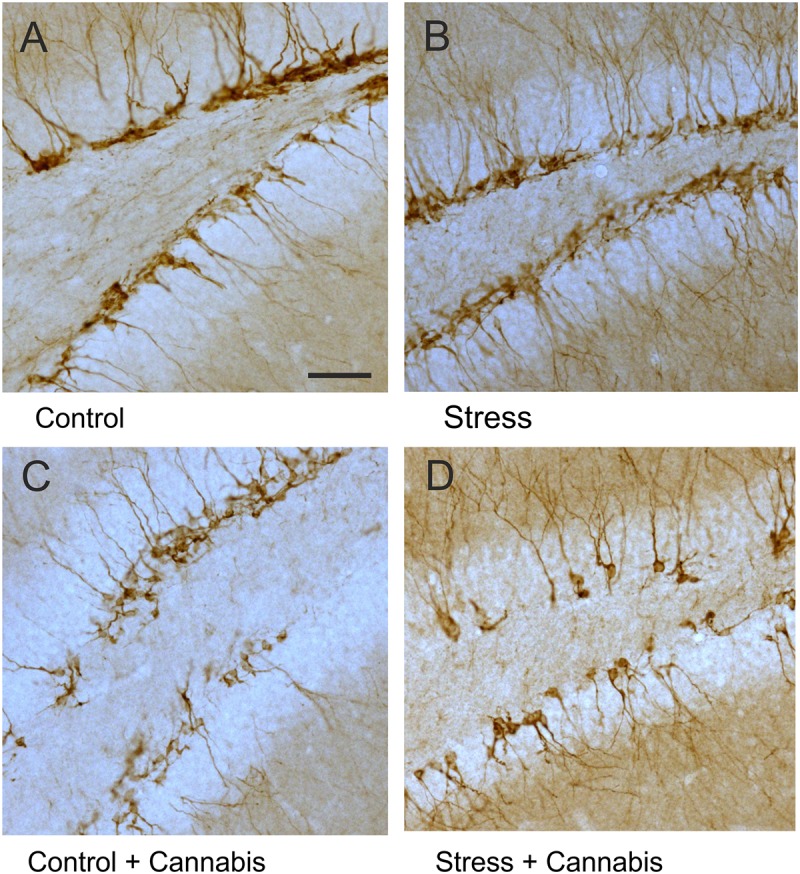
DCX-positive immature neurons in the dentate gyrus. Representative images demonstrating DCX-immunohistochemistry staining in the dentate gyrus of animals from the different treatment groups **(A–D)**. Note that DCX-positive cells were often disarrayed in the dentate gyrus of cannabis treated mice **(C,D)**. Scale bar: 50 μm for all images.

**FIGURE 8:**
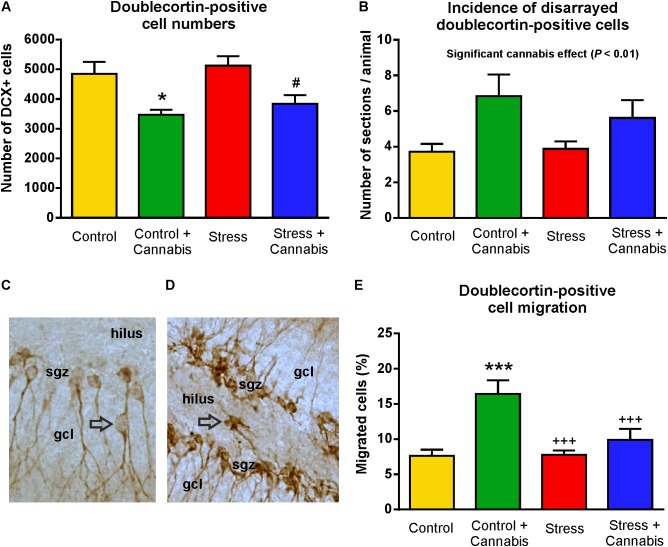
Marijuana smoke exposure had a pronounced effect on the number, migration and morphology of doublecortin-positive immature neurons. **(A)** Cannabis treatment significantly reduced the number of DCX-positive neurons in the dentate gyrus. Two-way ANOVA (stress × cannabis treatment) revealed a highly significant main effect of cannabis treatment (*P* < 0.001). Tukey’s *post hoc* test revealed further differences between the Control and Control + Cannabis groups (^∗^*P* < 0.05) and between the Stress and Stress + Cannabis treated groups (#*P* < 0.05). **(B)** The number of sections/animal in which we observed disarranged DCX-positive cells. Two-way ANOVA (stress × cannabis treatment) revealed a significant main effect of cannabis treatment (*P* < 0.01). **(C)** In control mice, the majority of the DCX+ immature neurons were located in the subgranular zone (sgz) and a small percentage, i.e., <5% of the cells, migrated either to the granule cell layer (gcl) or a few cells were found in the hilus **(D)**. This ratio was significantly altered by the cannabis exposure. **(E)** In the marijuana treated animals >15% of the DCX+ cells were found in the granule cell layer, hilus or occasionally in the molecular layer. Two-way ANOVA (stress × cannabis treatment) revealed a significant main effect of stress (*P* < 0.05) and cannabis treatment (*P* < 0.001), and a significant interaction between the factors (*P* < 0.05). Tukey’s *post hoc* test found significant differences between the groups: ^∗∗∗^*P* < 0.001 *versus* Control; +++*P* < 0.001 *versus* the Control + Cannabis treated group.

The population of immature neurons was visualized with DCX-immunohistochemistry (Brown et al., 2003; Rao and Shetty, 2004, **Figure 7**). Stress had no effect, but cannabis treatment significantly reduced the number of DCX-positive neurons in the dentate gyrus (**Figure 8A**). Two-way ANOVA (stress × cannabis treatment) revealed a highly significant main effect of cannabis treatment [*F*(1,32) = 18.86, *P* = 0.0001]. Tukey’s *post hoc* test revealed further differences between the Control and Control + Cannabis (*q* = 4.50, *P* < 0.05) and between the Stress and Stress + Cannabis treated groups (*q* = 4.19, *P* < 0.05) (**Figure 8A**).

**FIGURE 9:**
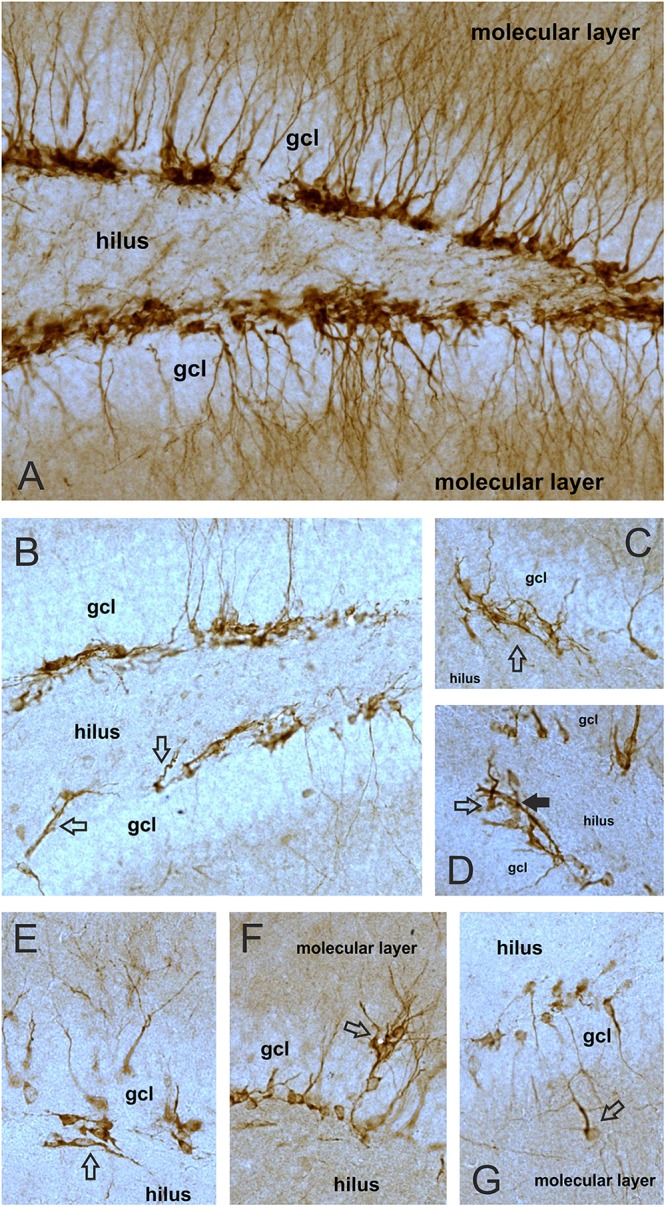
Long-term cannabis exposure alters the morphology of DCX-positive neurons. Representative images demonstrating DCX+ immature neurons with normal appearance in a control animal **(A)** and cells with abnormal morphology in cannabis treated mice **(B–G)**. **(A)** In control mice almost all the DCX+ neurons were located in the subgranular zone, i.e., in a cell layer between the hilus and granule cell layer (gcl). Normal DCX+ cells had DCX-expressing dendrites projecting through the gcl and arborized in the molecular layer. The principal dendrites were typically running parallel to each other in the gcl giving an orderly, well-aligned appearance to the neurons. **(B)** DCX+ neurons in the cannabis treated animals often lost their dendritic DCX-expression. Arrows point to neurons with abnormal dendritic projections into the hilus. **(C)** DCX+ neurons with dendrites projecting into all directions, giving a disorganized, chaotic appearance to the neurons. **(D)** Open arrow points to a neuron with basal dendrites, i.e., with dendritic projections into the hilus. Black arrow points to a cell with bipolar appearance. **(E)** A bipolar DCX+ neuron in the hilus with dendritic projections running parallel to the gcl. **(F)** Neurons migrating out of the gcl into the molecular layer. **(G)** An ectopic DCX+ neuron with basal dendrite that migrated completely out of the gcl into the molecular layer. All images were taken with the same magnification (20× objective).

During the quantitative analysis of the DCX+ neurons we noticed that in the dentate gyrus of cannabis treated mice, DCX+ cells often had an unusual or abnormal appearance (**Figure 9**). For example, cannabis treatment significantly altered the dendritic morphology of the DCX+ cells. Many DCX+ cells lost their dendritic DCX-expression (**Figure 9B**) and occasionally we observed bipolar DCX+ neurons (**Figure 9E**), or cells with basal dendrites (**Figures 9B,G**). In order to quantify these morphological changes of the DCX+ cells, we quantified the number of sections/animal where we could find DCX+ neurons with abnormal appearance (**Figure 8B**). Two-way ANOVA (stress × cannabis treatment) revealed that cannabis treatment had a significant main effect [*F*(1,32) = 8.35, *P* = 0.01] indicating that the incidence of DCX+ cells with abnormal appearance was significantly higher in the cannabis treated animals (**Figure 8B**).

We also quantified cell migration of the DCX+ cells. In control mice, the majority, i.e., >90% of the DCX+ immature neurons, were located in the germinative subgranular zone (sgz). A small percentage, i.e., <5% of the cells, migrated out of the sgz either to the granule cell layer (gcl) (**Figure 8C**), or some cells were also found in the hilus (**Figure 8D**). This ratio was significantly altered by the marijuana exposure. In the cannabis treated animals, >15% of the DCX+ cells were found outside of the sgz (**Figure 8E**). Two-way ANOVA (stress × cannabis treatment) yielded a highly significant main effect of cannabis treatment [*F*(1,32) = 15.87, *P* < 0.001], a significant main effect of stress [*F*(1,32) = 5.46, *P* < 0.05], and a significant interaction between the two factors [*F*(1,32) = 5.96, *P* < 0.05]. Furthermore, Tukey’s *post hoc* test revealed significant differences between the Control and Control + Cannabis (*q* = 6.42, *P* < 0.001), Stress *versus* Control + Cannabis treated groups (*q* = 6.32, *P* < 0.001), and between the Stress + Cannabis and Control + Cannabis treated groups (*q* = 4.78, *P* < 0.01) (**Figure 8E**).

**FIGURE 10:**
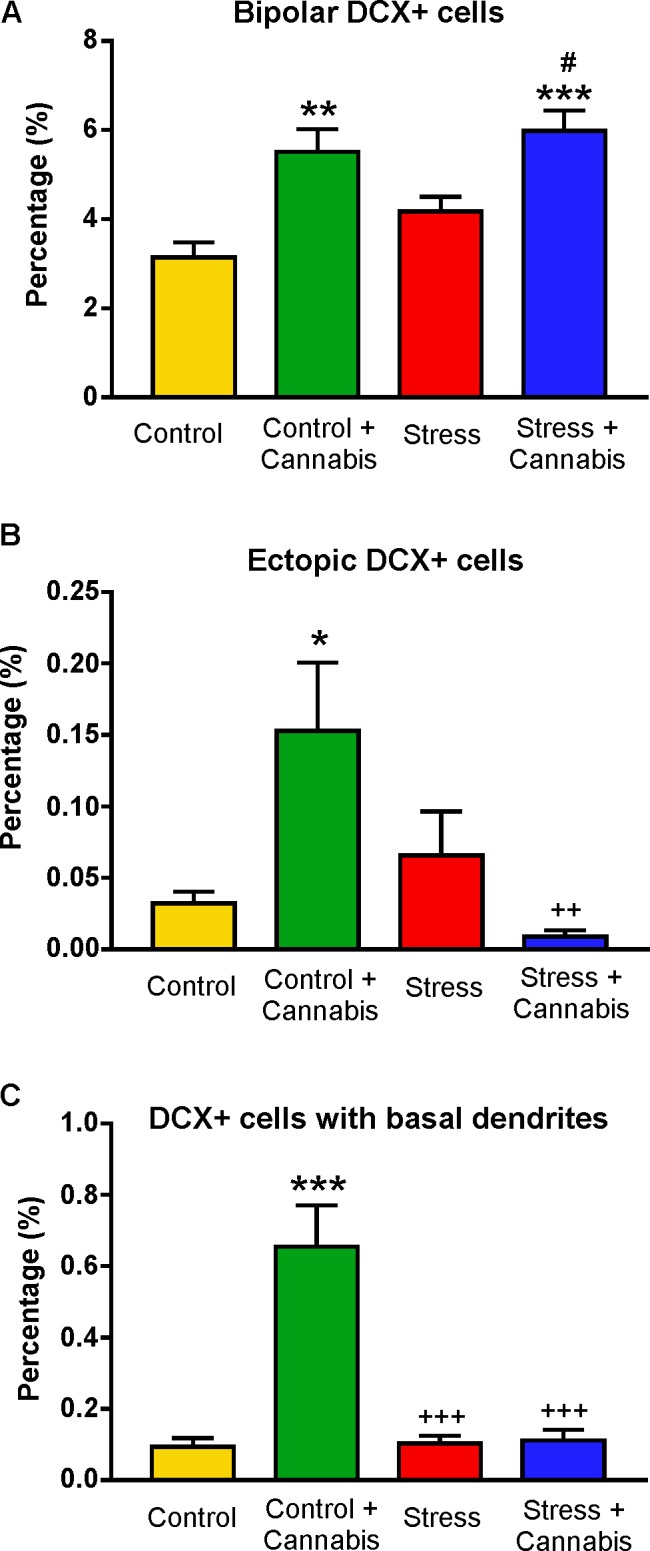
The incidence of doublecortin-positive neurons with unusual or abnormal morphology. **(A)** Cannabis treatment significantly increased the percentage of bipolar DCX+ cells. Two-way ANOVA (stress × cannabis treatment) revealed a highly significant main effect of cannabis treatment (*P* < 0.001). Tukey’s *post hoc* test revealed further significant group differences: ^∗∗^*P* < 0.01 *versus* Control; ^∗∗∗^*P* < 0.001 *versus* Control; #*P* < 0.05 *versus* Stress. **(B)** Cannabis treatment significantly increased the percentage of ectopic DCX+ cells, i.e., DCX+ cells which were found in the molecular layer of the dentate gyrus (see **Figure 9G**). Statistics: Two-way ANOVA followed by Tukey’s *post hoc* test. ^∗^*P* < 0.05 *versus* Control; ++*P* < 0.01 *versus* the Control + Cannabis treated group. **(C)** Cannabis treatment significantly increased the incidence of DCX+ cells with basal dendrites. Statistics: Two-way ANOVA followed by Tukey’s *post hoc* test. ^∗∗∗^*P* < 0.001 *versus* Control; +++*P* < 0.001 *versus* the Control + Cannabis treated group.

Quantitative data on the incidence of DCX+ cells with abnormal appearance is shown on **Figure 10**. Cannabis treatment significantly increased the percentage of bipolar DCX+ cells. Two-way ANOVA (stress × cannabis treatment) yielded a highly significant main effect of cannabis treatment [*F*(1,32) = 41.36, *P* < 0.001], and Tukey’s *post hoc* test revealed significant differences between the Control and Control + Cannabis (*q* = 5.75, *P* < 0.01), Control *versus* Stress + Cannabis groups (*q* = 6.90, *P* < 0.001), and between the Stress and Stress + Cannabis groups (*q* = 4.26, *P* < 0.05) (**Figure 10A**). Cannabis treatment also increased the frequency of ectopic DCX+ cells, i.e., the incidence of cells that were found in the molecular layer of the dentate gyrus (**Figures 9G**, **10B**). The number of DCX+ neurons with basal dendrites was also much higher in the cannabis treated animals (**Figure 10C**). Two-way ANOVA (stress × cannabis treatment) revealed a highly significant main effect of cannabis treatment [*F*(1,32) = 22.45, *P* < 0.001], a significant main effect of stress [*F*(1,32) = 19.75, *P* < 0.001], and also a significant interaction between the two factors [*F*(1,32) = 21.10, *P* < 0.001]. Furthermore, Tukey’s *post hoc* test revealed further significant group differences between the Control and Control + Cannabis treated groups (*q* = 8.87, *P* < 0.001), between the Stress and Control + Cannabis groups (*q* = 8.47, *P* < 0.001), and between the Control + Cannabis and Stress + Cannabis treated groups (*q* = 8.47, *P* < 0.001).

## Discussion

The principal aim of the present study was to model regular marijuana consumption of humans in an experimental setting. Since most of the users are young people who claim that they consume cannabis to relax from everyday stress therefore, we exposed young experimental mice to daily stress and concomitant cannabis smoke. While most experimental studies inject synthetic cannabinoids, we decided to use marijuana smoke exposure, because smoking is the most typical route of application in humans. Furthermore, we did a long-term, repeated exposure for 8 weeks, using daily restraint stress for 6 h/day and exposing animals to the smoke of 4 “joints”/day during the end of the restraint stress. Our results demonstrate that both stress and cannabis exposure significantly reduced body weight gain of the animals. However, cannabis could alleviate the stress-induced reduction of body-weight gain. We expected to see marijuana-induced changes in pulmonary functions, but instead it was the chronic stress which inhibited the maturation of most of the lung functions. In the behavioral tests measuring the cognitive performance of the animals, neither stress, nor cannabis treatment had any effect after 3 or 4 weeks of exposure. In the OFT, where we evaluated the anxiety related behavior of the animals after 8 weeks of treatment, stress had an anxiogenic effect, while cannabis had only a mild tendency to reverse the stress-induced anxiety. Marijuana however had a strong effect on self-grooming. Cannabis treated mice started to groom themselves much sooner than controls and also spent significantly more time with grooming. Finally, we examined adult hippocampal neurogenesis and we found that chronic stress – as expected – blocked progenitor cell proliferation in the dentate gyrus, but marijuana smoking had no influence on that. In contrast to that, cannabis smoke exposure had a pronounced effect on the doublecortin-positive immature neurons. Cannabis significantly reduced the number of DCX+ neurons in the dentate gyrus, stimulated their migration and the dendritic morphology of the cells was also profoundly altered by the marijuana treatment. In sum, with this experimental design we found that long-term exposure to cannabis smoke had either no effect or negative impact on various health-related measures.

Cannabis contains over 500 different compounds (ElSohly and Gul, 2014), but the potency of marijuana is usually judged based on the Δ9-THC content of the preparation. We did a HPLC analysis to assess the major components of the cannabis smoke which was used in this experiment. Results of this analysis revealed that the Δ9-THC content was 10-fold higher than its CBD content (**Table 1**). This difference was even more pronounced in the urine samples, where the Δ9-THC content was 30-fold higher than the CBD content. Several reports indicate that the potency of marijuana sold on the streets has increased dramatically over the past few decades (Mehmedic et al., 2010; ElSohly et al., 2016) and it has been suggested that this increase in potency has been the reason for the rising emergency department visits involving marijuana use in the US (Data Spotlight, 2012).

Our principal aim was to mimic the regular cannabis consumption of humans. However, it is very difficult to define the average frequency of cannabis use in the general population. A recent US survey documented that 3–4% of all primary care patients in Washington State report on a daily use, and 10–14% of them consume cannabis at least once a week or month (Lapham et al., 2017). Young adults (aged 18–29 years) use cannabis much more frequently and 6–12% of them report to consume it on a daily basis and 25–30% of them use it at least once a week or month (Lapham et al., 2017). US citizens, who consume cannabis regularly, report to use an average of 9.4 ± 9.7 joints weekly (1–60 joints/week) (Goodwin et al., 2008). From this perspective the regimen of cannabis exposure that we used, i.e., 4 joints/day (20 joints/week) was rather strong. Furthermore, we determined the THC/CBD/CBN and carboxy-THC concentrations in the urine of the mice. Our present cannabis treatment protocol resulted in a low (2 ng/mL) urine concentration of carboxy-THC. According to the guidelines of the Mayo Clinic, the presence of carboxy-THC in human urine samples at concentrations > 15.0 ng/mL is a strong indicator that the patient has used marijuana. The presence of carboxy-THC in urine > 100.0 ng/mL indicates relatively recent use, probably within the past 7 days. Levels of >500.0 ng/mL suggest chronic and recent use^1^. In this comparison, our cannabis exposure regimen was modest. However, it should be emphasized that the drug metabolism rate of mice is much higher compared to humans. We are not aware of any study that evaluated the amount of marijuana smoke exposure that is necessary to reach serum or urine concentrations of THC/CBD/CBN and carboxy-THC comparable to the human samples. It has been documented that rats have to be exposed to the smoke of 60 cigarettes/day to reach serum levels of nicotine and cotinine that is comparable to human cigarette smokers (Small et al., 2010; Bruijnzeel et al., 2011). However, to apply such a high dose would be technically impossible for us and also we would not get an ethical permission to expose animals to e.g., 60 joints/day over a 2-month period.

The clinical findings describing the effects of marijuana on the body and mind are often conflicting. For example, cannabis is known to stimulate appetite and potentially promote weight gain in patients with cancer of HIV (Abrams and Guzman, 2015; Kramer, 2015) whereas, the large epidemiological studies involving the general population consistently report that users of marijuana tend to have lower body mass indices (Sansone and Sansone, 2014). Therefore, it is important that the well-controlled experiments should aim to mimic real life situations as much as possible. In our experiment, both cannabis smoke and stress reduced the body weight gain of the animals, and in this case we found a significant interaction between these two factors. This means that cannabis treatment could alleviate the stress-induced inhibitory effect on body weight gain.

Marijuana smoke contains many of the same toxins and carcinogens as tobacco smoke and thus, irritates the respiratory system similarly to tobacco. Clinical studies indicate that regular cannabis smoking alone is associated with airway inflammation (Jett et al., 2018). In our recent experimental study, daily marijuana inhalation for 4 months resulted in inflammation, tissue destruction, and emphysema (Helyes et al., 2017). In the present study, cannabis smoke did not affect any of the pulmonary functions. Notably, a large clinical study in the US could not find any adverse spirometric changes with cumulative lifetime marijuana use of up to 20 joint-years either (Kempker et al., 2015). In our present study, it was the repeated stress exposure that inhibited the development of several pulmonary functions. To our best of knowledge, ours is the first study to reveal such negative effect of chronic stress on pulmonary functions. We could not find any comparable experimental data in the literature. However, human studies document that experiencing psychological stress in children is significantly associated with asthma morbidity (Chen et al., 2006). Preclinical studies reported that chronic stress in mice can result in pneumonia (Kiank et al., 2008) and can worsen allergic airway inflammation (Okuyama et al., 2007).

Several research groups investigated the behavioral effects of marijuana smoke or specific cannabinoid molecules in the OFT (e.g., Bruijnzeel et al., 2016; Murphy et al., 2017; Tomas-Roig et al., 2018). These studies report on mixed results. Repeated daily injection of Δ9-THC into adolescent or adult CD1 mice had no behavioral consequences in the OFT (Murphy et al., 2017). Injection of the CB1/CB2 receptor agonist WIN 55,212-2 to C57Bl6/J mice resulted in reduced locomotor activity, but had no effect on distance traveled in the center of the open field (Tomas-Roig et al., 2018). In rats, acute cannabis smoke exposure induced a brief increase in locomotor activity which was then followed by a prolonged decrease in locomotor activity and rearing (Bruijnzeel et al., 2016). In our study, cannabis smoke exposure had no effect on locomotor activity or on anxiety-related behavior. Marijuana smoke also did not alter the anxiogenic effect of repeated stress. However, cannabis smoke had a pronounced effect on self-grooming. Both stressed and cannabis treated mice spent significantly more time with self-grooming and they also started to groom themselves significantly sooner than control mice. Self-grooming is a complex innate behavior of rodents which is regulated by several neural circuits. Aberrant self-grooming has been described in animal models of several neuropsychiatric disorders including substance abuse (Kalueff et al., 2016). The same review also suggested that self-grooming has a great value for translational neuroscience, since each disease model may result in a distinct grooming phenotype (Kalueff et al., 2016). Our present study supports the notion that the microstructure of self-grooming should be investigated in more detail, because abnormal grooming behavior could signal the stress or anxiety level of the animals in the specific models (Kalueff et al., 2007).

The effect of cannabis use on the cognitive performance of the individual is probably the most ambiguous issue. Recent data suggest that cannabis use can improve various cognitive and executive functions (Osborne et al., 2017; Tervo-Clemmens et al., 2018; Gruber et al., 2018), while others claim that it impairs cognition (Volkow et al., 2014, 2016; Broyd et al., 2016; Curran et al., 2016). In this experiment, we could not detect any (positive or negative) effect of marijuana smoke exposure on the cognitive performance of the animals in the novel object recognition and Y maze tests. It has been argued that a potential explanation for the conflicting data in the literature is the different ratio of Δ9-THC and CBD content in the marijuana samples used in the experiment (Fadda et al., 2004). Data suggests that high Δ9-THC or low CBD cannabis results in greater cognitive impairment (Colizzi and Bhattacharyya, 2017; Murphy et al., 2017). In our case, the cognitive tests were done at the end of the 3rd and 4th week (**Figure 1**) and it might be that the cannabis exposure was too short at these time points to result in a detectable difference. Probably, it might have been better to do the cognitive testing at the end of the experiment which would have also allowed us to directly compare the cognitive performance of the animals with the findings on adult neurogenesis. The reason why we did not do the cognitive testing at the end of the experiment was that it is known that learning can affect the process of adult neurogenesis (Gould et al., 1999) and we wanted to avoid too many testing at the end of the experiment that could interfere with each other and with neuroplasticity.

Neurogenesis in the adult hippocampal dentate gyrus is a special form of neuroplasticity that has been implicated in various physiological and pathophysiological conditions including substance abuse (Eisch et al., 2000; Mandyam et al., 2008; Noonan et al., 2010; Bayer et al., 2015). It is well documented that the endocannabinoid system and the CB1 cannabinoid receptor mediate neural progenitor cell proliferation and neurogenesis (e.g., Aguado et al., 2005, 2007). Numerous studies investigated the effect of cannabinoids on adult hippocampal neurogenesis (reviewed recently by Prenderville et al., 2015), but to our best of knowledge, none of these studies used cannabis smoke exposure, instead they all injected synthetic cannabinoids or plant-derived extracts to the experimental animals. There have been numerous reports on a positive, stimulatory effect of cannabinoid treatment on adult neurogenesis (Jiang et al., 2005; Palazuelos et al., 2006; Marchalant et al., 2009; Wolf et al., 2010; Rivera et al., 2011; Suliman et al., 2018). Furthermore, several reports suggested that facilitation of the cannabinoid signaling in the hippocampus may prevent stress-induced behavioral changes (Campos et al., 2013; Scarante et al., 2017; Fogaça et al., 2018). In our present study, chronic stress – as expected – reduced cell proliferation in the dentate gyrus, but cannabis smoke had no influence on this effect of stress. However, marijuana smoke had a distinct effect on the DCX+ cells, by reducing their number and affecting their morphology and migration. In the literature, one can find controversial data on the influence of cannabinoids on DCX+ neurons. In non-stressed Swiss mice, repeated administration of CBD at a lower dose (3 mg/kg) increased the number of DCX+ cells, but at a higher dose (30 mg/kg) it had a negative effect (Schiavon et al., 2016). Another study demonstrated that when CBD was fed to female C57Bl/6 mice it stimulated adult neurogenesis, whereas Δ9-THC had no such effect (Wolf et al., 2010). Yet, more recently, it was shown that injection of Δ9-THC to rats can stimulate adult neurogenesis (Suliman et al., 2018). Another study reported that daily injection of the cannabinoid agonist WIN 55,212-2 for 3 weeks to adolescent rats reduced the number of newly generated neurons in hippocampus (Abboussi et al., 2014).

To our best of knowledge, so far there was only one research group which carried out experiments where stress was combined with cannabinoid treatment and the consequences on behavior and hippocampal neuroplasticity were investigated. Guimarães and co-workers used chronic unpredictable stress (CUS) in combination with repeated injection of pure CBD and reported on a pronounced anxiolytic effect of CBD treatment which could also normalize the stress-induced inhibition of hippocampal progenitor cell proliferation and neurogenesis, i.e., it reversed the stress-induced reduction of BrdU- and DCX-positive cell numbers (Campos et al., 2013). In their more recent study, they replicated and extended these findings by demonstrating that CBD treatment can prevent the stress-induced inhibitory effects on adult neurogenesis via the activation of CB1/CB2 receptors (Fogaça et al., 2018). Our present experiment could not replicate these findings. There are several possible explanations for this: (1) The Guimarães group used a different stress protocol which lasted only for 14 days (Campos et al., 2013; Fogaça et al., 2018). It might be, that our stress protocol was too aggressive and/or lasted too long, as it was 4× longer than the protocol of the Guimarães group. (2) The Guimarães group treated their animals with pure CBD, whereas in our case the marijuana smoke included hundreds of molecules and had high Δ9-THC content (10:1 ratio of Δ9-THC : CBD content). Thus, it might be that in our case the high Δ9-THC content concealed the beneficial effects of the CBD.

In the present experiment, marijuana reduced the number DCX+ cells, stimulated their migration and significantly altered their dendritic architecture. Future investigations are needed to determine whether these cellular changes should be considered as positive or negative effects. Previous studies reported that CBD application can increase DCX+ cell numbers in various experimental conditions (Schiavon et al., 2016; Mori et al., 2017). Another study comparing the effects of Δ9-THC with CBD found that Δ9-THC can reduce DCX+ cell numbers (Wolf et al., 2010, but see also Suliman et al., 2018). High Δ9-THC content of our sample might explain our finding on reduced DCX+ cell numbers. Other research group also documented that CBD treatment can stimulate DCX+ cell migration from the subgranular zone to the granular zone of dentate gyrus (Fogaça et al., 2018). This finding is in harmony with the knowledge that endocannabinoids play an important role during brain development regulating cell proliferation, migration, differentiation and survival (Harkany et al., 2007). A recent developmental study provided further evidence that prenatal exposure to the CB1/CB2 receptor agonist WIN 55,212-2 can alter the migration of early-born neurons in the cerebral cortex (Saez et al., 2014). To our best of knowledge our present data is the first to report on altered dendritic architecture of DCX+ cells in response to cannabinoid treatment. Normal DCX+ cells have DCX-expressing dendrites which project through the granule cell layer to the molecular layer. In contrast to that, we observed numerous DCX+ cells which lost their dendritic DCX-expression in the marijuana treated animals. Furthermore, in the cannabis treated animals, we observed an increased occurrence of bipolar DCX+ cells and cells with basal dendrites. It is known from the literature that basal dendrites on dentate granule cells can be induced by epileptic seizures (Ribak et al., 2012; Hester and Danzer, 2014) thus, the presence of such cells suggests abnormal neuronal circuitry, or cellular niche. We also found higher frequency of ectopic DCX+ cells in the cannabis treated animals. One possible explanation for the fact that previous studies did not report on such abnormal looking cells after cannabinoid treatment is that our analysis was much more careful and thorough compared to the previous studies. Most studies examine only 5–6 sections/animal, whereas we examined every 3rd sections from the serially cut hippocampus, yielding in average 25 analyzed sections/animal.

However, the present study has some limitations. As we already discussed, it is very difficult to find a marijuana dosage that is truly comparable to the human situation. Human consumption habits are highly variable and mice have a much higher metabolic rate compared to humans which makes it very difficult to reach comparable serum or urine concentrations of cannabinoid metabolites. Not to mention the fact that street marijuana samples have highly variable potency and efficacy. We cannot rule out the possibility that using a higher dose of cannabis, or a different sample having a different potency/efficacy could result in a different outcome in a similar experimental setting. We do not question that treatment with CBD has a positive effect in stressed animals as it has been well documented by others (e.g., Campos et al., 2013; Fogaça et al., 2018). Furthermore, employing other type of stress protocols may also yield different results. It is possible that in our study the stress procedures were too severe for the animals and that was the reason why cannabis treatment could not alleviate the stress-induced effects.

To our best of knowledge, this is the first experimental study to combine chronic stress exposure with concomitant cannabis smoke inhalation. We report here that long-term exposure to these factors can influence several health-related measures, but our present experimental design could not reveal any significant interaction between these two factors (except for body weight gain). We report here for the first time, that chronic cannabis smoke can significantly alter the morphology of DCX+ neurons in the dentate gyrus and this finding deserve further investigations. In the present experiment, chronic cannabis smoke had either no effect or negative impact on the physical and mental condition of the animals. We should however, emphasize that modeling cannabis smoke consumption in experimental animals is a difficult issue, because (1) human smoking habits can be extremely variable, (2) rodents have a much higher metabolic rate, therefore, it is very difficult to apply cannabis smoke in dosage that results in a serum or urine concentrations of cannabinoid metabolites that are comparable to humans. Finally, the present study cannot rule out the possibility that application of different doses of cannabis smoke or pure CBD treatment may yield a different outcome in a similar experimental setting.

## Author Contributions

BC, ZH, and KC had the experimental concepts and designed the experiments. KR did the behavioral tests, DCX- immunohistochemistry and all cell counting, and prepared the figures for the paper. KC carried out the treatment procedures, behavioral tests, and data analysis. ZV analyzed the behavioral data of the NOR and OFTs. DC did the BrdU-immunohistochemistry. ÁB did the HPLC analysis on the cannabis smoke. MM and ZK measured metabolites in the urine samples. All authors contributed to the writing of the paper and/or revising it critically for important intellectual content. All authors approved the final version to be published and agreed to be accountable for all aspects of the work in ensuring that questions related to the accuracy or integrity of any part of the work are appropriately investigated and resolved.

## Conflict of Interest Statement

The authors declare that the research was conducted in the absence of any commercial or financial relationships that could be construed as a potential conflict of interest.

## Footnotes

1 https://www.mayomedicallaboratories.com/test-catalog/Clinical+and+Interpretive/8898
